# Increased trunk fat is associated with altered gene expression in breast tissue of normal weight women

**DOI:** 10.1038/s41523-021-00369-8

**Published:** 2022-01-27

**Authors:** Byuri Angela Cho, Neil M. Iyengar, Xi Kathy Zhou, Hillary Mendieta, Lisle Winston, Domenick J. Falcone, Jonathan Landa, Monica Morrow, Andrew J. Dannenberg

**Affiliations:** 1 Department of Medicine, Weill Cornell Medical College, New York, NY USA; 2 Department of Medicine, Memorial Sloan Kettering Cancer Center, New York, NY USA; 3 Department of Population Health Sciences, Weill Cornell Medical College, New York, NY USA; 4 Department of Pathology and Laboratory Medicine, Weill Cornell Medical College, New York, NY USA; 5 Department of Radiology, Memorial Sloan Kettering Cancer Center, New York, NY USA; 6 Department of Surgery, Memorial Sloan Kettering Cancer Center, New York, NY USA

**Keywords:** Breast cancer, Cancer genomics

## Abstract

Increased trunk fat is associated with an elevated risk of breast cancer in normal-weight postmenopausal women. The main objective of this study was to determine whether levels of trunk fat are associated with changes in breast gene expression in normal-weight women. Non-tumorous breast tissue was collected from 32 normal BMI women who underwent mastectomy for breast cancer risk reduction or treatment. Body composition was measured by dual-energy x-ray absorptiometry. High levels of trunk fat were associated with a large number of differentially expressed genes and changes in multiple pathways and processes potentially linked to breast cancer pathogenesis. High levels of trunk fat were also associated with an elevated immune score and increased levels of leptin, *CCL2*, *VEGF-C*, *IL6*, and aromatase. Collectively, these results help to explain why high levels of trunk fat are associated with an increased risk of breast cancer in normal BMI women.

## Introduction

Obesity is a risk factor for numerous cancers, including postmenopausal hormone receptor-positive breast cancer^1–3^. The link between excess body fat and malignancy has largely been established using anthropometric measurements such as body mass index (BMI), which takes into account weight and height but does not discriminate between muscle, bone, and fat. Some women deemed to be healthy because of a normal BMI have underlying cardiometabolic abnormalities and are commonly referred to as metabolically obese normal weight (MONW)^4^. Dual-energy x-ray absorptiometry (DXA) is used to obtain direct measures of body fat including trunk fat. In normal BMI women, high levels of body fat including trunk fat have been associated with enlarged breast adipocytes and breast white adipose tissue inflammation (B-WATi)^5^, as defined by the presence of crown-like structures (CLSs). B-WATi, in turn, has been associated with an elevated risk of breast cancer in women who have benign breast disease^6^. Given this background, we previously posited that normal BMI women with high levels of body fat would be at increased risk for postmenopausal breast cancer^7^. In a study of more than 3000 normal BMI women who had enrolled in the Women Health Initiative, we relied on DXA-derived measures of body fat and found that women in the top two quartiles of trunk fat had about a doubling in the risk of estrogen-dependent breast cancer compared to women in the lowest quartile of trunk fat^8^. The sample size was too small to determine whether the amount of trunk fat was also associated with altered risk of either HER-2/neu-overexpressing or triple-negative breast cancer. Circulating levels of C-reactive protein, insulin, leptin, and triglycerides were higher, whereas levels of sex hormone-binding globulin and high-density lipoprotein cholesterol were lower in those in the highest vs. lowest quartiles of trunk fat mass^8^. More recently, we found that high levels of blood C-reactive protein and testosterone or low blood levels of sex hormone-binding globulin were associated with an elevated risk of postmenopausal breast cancer among normal BMI women^9^.

It has become increasingly clear that normal BMI as a category includes women with relatively high levels of body fat who are at increased risk for postmenopausal breast cancer and that abnormal levels of systemic factors may be contributory^4,10^. Increased levels of aromatase, the rate-limiting enzyme for estrogen biosynthesis, have been reported in the breast tissue of normal BMI women with B-WATi and higher levels of trunk fat^5,7^. Despite these recent advances, very little is known about the molecular changes that occur in the breast itself in normal BMI women with high vs. low levels of trunk fat. Accordingly, the main objective of the current study was to use RNA-sequencing (RNA-seq) to define the changes in the breast transcriptome that occur in normal-weight women who have high vs. low levels of trunk fat.

## Results

### Study population

Subjects were recruited from April 2016 through August 2018 and underwent DXA scans prior to mastectomy^5^. RNA-seq data were obtained from non-tumorous breast tissue of 32 normal-weight women, which defined the study cohort. To determine the potential association between levels of trunk fat and breast gene expression, the 32 women were divided into two groups based on percent trunk fat and are referred to as high and low groups, respectively. A comparison was done of 16 normal-weight women with high levels of trunk fat (equal to or higher than the median of 29.45%) vs. 16 normal-weight women with low levels of trunk fat (less than the median of 29.45%). Women with high levels of trunk fat were older (*P* = 0.01), had a higher BMI (*P* = 0.003), were more likely to have B-WATi (*P* < 0.001) and had larger breast adipocytes (*P* < 0.001) (Table 1).

**Table 1:** Clinical features of normal weight women with high vs. low trunk fat.

Variables	All (*n* = 32)	Trunk fat low (*n* = 16)	Trunk fat high (*n* = 16)	*P* value
Age				
Median (IQR)	44.5 (39.5, 49.25)	40.5 (32, 47.25)	47 (44, 51.5)	0.011
Race, *n* (%)				
White	21 (67.74%)	12 (75%)	9 (60%)	0.12
Asian	6 (19.35%)	1 (6.25%)	5 (33.33%)	
Black or African American	2 (6.45%)	1 (6.25%)	1 (6.67%)	
Other	2 (6.45%)	2 (12.5%)	0 (0%)	
Unknown	1 (3.12%)	0 (0%)	1 (6.25%)	1
BMI				
Median (IQR)	21.8 (20.1, 22.63)	20.5 (19.67, 21.93)	22.4 (21.75, 23.42)	0.003
Menopausal, *n* (%)				
Post	6 (20%)	1 (6.67%)	5 (33.33%)	
Pre	24 (80%)	14 (93.33%)	10 (66.67%)	0.169
Unknown	2 (6.25%)	1 (6.25%)	1 (6.25%)	1
CLS, *n* (%)				
No	22 (68.75%)	16 (100%)	6 (37.5%)	
Yes	10 (31.25%)	0 (0%)	10 (62.5%)	<0.001
Adipocyte diameter (µ)				
Median (IQR)	89.32 (79.48, 100.16)	79.1 (72.78, 87.24)	97.14 (89.86, 106.15)	<0.001
Invasive, *n* (%)				
No	8 (25%)	5 (31.25%)	3 (18.75%)	
Yes	24 (75%)	11 (68.75%)	13 (81.25%)	0.685
HTN, *n* (%)				
No	30 (93.75%)	16 (100%)	14 (87.5%)	
Yes	2 (6.25%)	0 (0%)	2 (12.5%)	0.484
DM, *n* (%)				
No	32 (100%)	16 (100%)	16 (100%)	
Yes	0 (0%)	0 (0%)	0 (0%)	1
Dyslipidemia, *n* (%)				
No	30 (93.75%)	16 (100%)	14 (87.5%)	
Yes	2 (6.25%)	0 (0%)	2 (12.5%)	0.484

*BMI* body mass index, *CLS* crown-like structure, *HTN* hypertension, *DM* diabetes mellitus.

### Excess body fat is associated with changes in the transcriptome in the breast

**Fig. 1:**
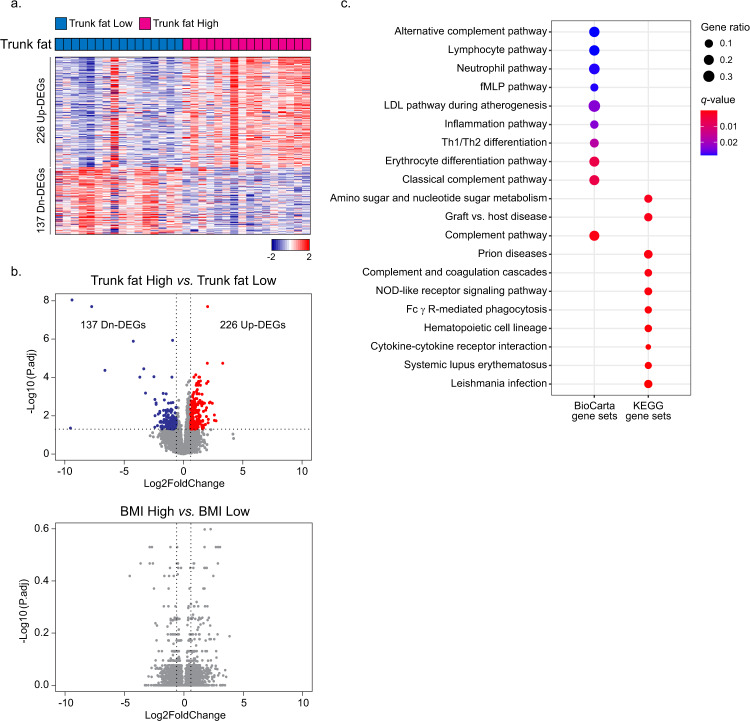
Transcriptome profiles of 32 women with normal BMI. **a** Heatmap demonstrating 226 upregulated and 137 downregulated differentially expressed genes (DEGs) from non-tumorous breast tissue of women with trunk fat high vs. trunk fat low. **b** Volcano plot illustrating the abundance of DEGs in (1) Trunk fat high vs. Trunk fat low and (2) BMI high vs. BMI low. Two vertical dashed lines correspond to −0.6 and 0.6, which are the Log_2_fold-change cutoffs for the DEGs. The horizontal dashed line corresponds to *P* adjusted value of 0.05, which is another cutoff for the DEG. **c** Biological pathway changes associated with increased trunk fat percent in normal BMI women. Gene ratio indicates the ratio of genes that are included within each pathway. *q* value is a *p* value that is adjusted for the false discovery rate. *q* < 0.05 is considered significant.

Breast tissue was obtained from women who either did or did not have tumors. If a tumor was present, to minimize the possibility that the tumor would influence the transcriptome of non-tumorous tissue, we evaluated tissue from an uninvolved quadrant of the breast. Principal component analysis of tissue from women with or without tumors suggested a homogeneous distribution of cases (Supplementary Fig. 1); hence, the presence of a tumor did not have a significant effect on the transcriptome of tissue from an uninvolved quadrant of the breast. Next, we investigated the effects of high vs. low trunk fat on the transcriptome in the non-tumorous breast. A total of 226 upregulated and 137 downregulated differentially expressed genes (DEGs) (P.adj <0.05, |Log_2_FoldChange| > 0.6) were found in high compared to low trunk fat women using DESeq2 (Fig. 1a and Supplementary Table 1). These effects are clearly seen in the heatmap and volcano plot shown in Fig. 1a and Fig. 1b (top panel), respectively. A similar analysis was carried out based on BMI. In this instance, high BMI was defined as equal to or higher than the median of 21.8 kg/m^2^, whereas low BMI was defined as <21.8 kg/m^2^. Interestingly, when comparing high vs. low BMI, there were no DEGs (Fig. 1b, bottom panel).

Increased trunk fat is associated with older age, larger breast adipocytes, and B-WATi. Hence, we posited that there would be an overlap between the DEGs associated with excess trunk fat and these other parameters. First, we divided the patients into two age groups using the age of 45 years, the median age of the cohort. In an analysis of those 45 years of age and above vs. less than 45 years of age, there were only one upregulated and three downregulated DEGs (Supplementary Table 2). In a comparison of postmenopausal vs. premenopausal women, three upregulated DEGs and nine downregulated DEGs were found (Supplementary Table 3). In contrast to age and menopause status, a comparison of nontumorous breast tissue from CLS(+) vs. CLS(−) cases and for large vs. small breast adipocytes led to the identification of numerous DEGs. In the case of CLS(+) vs. CLS(−) cases, there were 45 upregulated and 29 downregulated DEGs (Supplementary Table 4). Using the median value of adipocyte diameter (89.32 µ), the samples were divided into two groups: small adipocytes (<89.32 µ) and large adipocytes (> or =89.32 µ). 116 upregulated and 176 downregulated DEG were found (Supplementary Table 5). Next, we investigated whether there was an overlap between the DEGs for high vs. low trunk fat and the DEGs for BMI, menopause, CLS status, and adipocyte size. There was no overlap in upregulated or downregulated genes for trunk fat (%) vs. BMI or trunk fat (%) vs. menopausal status. By contrast, we did identify significant overlap between trunk fat (%) and upregulated (Supplementary Fig. 2a and Supplementary Tables 4 and 5) and downregulated genes (Supplementary Fig. 2b and Supplementary Tables 4 and 5) for CLS status and adipocyte size.

### Interpreting the global transcriptome changes in terms of biological pathways

**Fig. 2:**
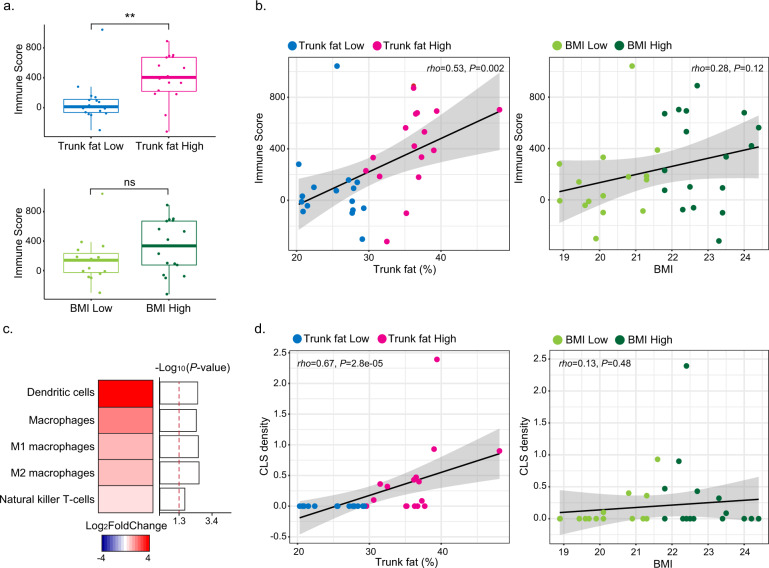
Immune cell population changes in association with high vs. low trunk fat in normal BMI individuals. **a** Immune score was calculated to observe overall immune cell population change with trunk fat and BMI. The difference was more significant when trunk fat was used as a parameter. For both trunk fat and BMI, the median value was used to divide the samples into two groups (*n* = 16/group). **P < 0.01; ns not significant. **b** Correlation between immune score vs. trunk fat percentage (left) or BMI (right). **c** Heatmap illustrating increased immune cell population in trunk fat high group when compared to the trunk fat low group. The red dashed line corresponds to *P* value of 0.05. **d** Correlation between CLS density vs. trunk fat percentage (left) or BMI (right). CLS density equals #CLS/cm^2^.

Both BioCarta and KEGG databases were used to classify the DEGs into relevant biological pathways. Numerous pathways were enriched with upregulated DEGs but not downregulated DEGs (Fig. 1c). Among the pathways in the non-tumorous breast that were enriched in association with increased trunk fat were the complement pathway, inflammation pathway, cytokine-cytokine receptor interaction, NOD-like receptor signaling pathway and graft-vs.-host disease. Collectively, these findings suggested that there were significant immunological alterations associated with high vs. low levels of trunk fat. Based on the above findings, we next used Estimate R package^11^, a computational approach, to determine an immune score. A significant increase in the immune score was found in association with high levels of trunk fat (Trunk fat Low vs. Trunk fat High) (*P* = 0.002) but not high BMI (BMI Low vs. BMI High; *P* = 0.18) (Fig. 2a). In fact, percent trunk fat positively correlated with the immune score (*rho* = 0.53, *P* = 0.002; Fig. 2b, left hand panel). The same was not true for BMI (rho = 0.28, *P* = 0.12; Fig. 2b, right hand panel). Additional studies were carried out to examine the relationship between percent trunk fat and immune cell populations. xCell was used to estimate immune cell populations and suggested increased macrophages (M1 and M2), dendritic cells, and natural killer T cells in association with high compared with low trunk fat (Fig. 2c). The increase in dendritic cells and macrophages associated with high vs. low trunk fat was confirmed using immune cell markers defined by Danaher et al.^12^ (Supplementary Fig. 3). To validate the increase in macrophages, CD68 immunohistochemistry was carried out and revealed a positive correlation between percent trunk fat and density of CLS (rho = 0.67, *P* = 2.8e−05) (Fig. 2d, left-hand panel). The same was not true for BMI (rho = 0.13, *P* = 0.48) (Fig. 2d, right hand panel). Levels of *CD68*, a macrophage marker, also showed a strong positive correlation with the estimated population of macrophages (rho = 0.93, *P* = 2.9e−14) (Supplementary Fig. 4).

**Fig. 3:**
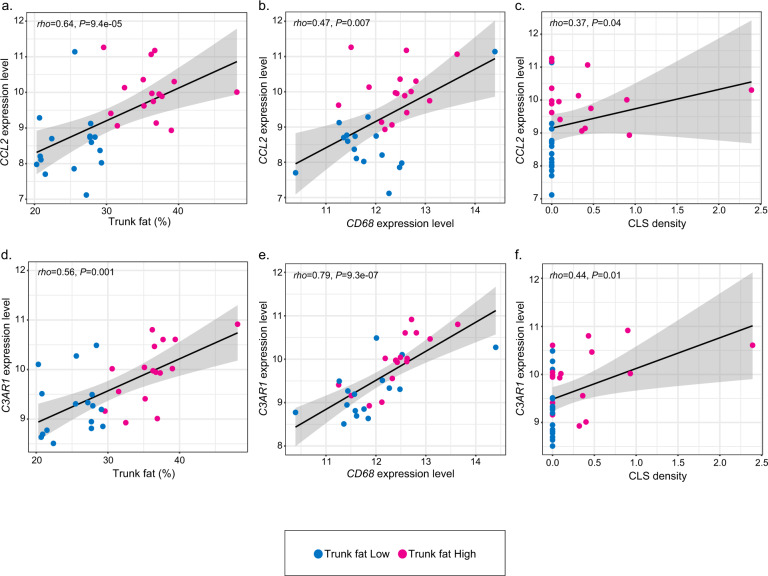
Correlations between the levels of expression of genes that have a role in white adipose inflammation, trunk fat%, and CLS density. **a** Correlation between trunk fat percent and *CCL2* expression level. **b** Correlation between levels of *CCL2* and *CD68*. **c** Correlation between *CCL2* expression level and CLS density. CLS density equals #CLS/cm^2^. **d** Correlation between trunk fat percent and *C3AR1* expression level. **e** Correlation between levels of *C3AR1* and *CD68*. **f** Correlation between *C3AR1* expression level and CLS density. CLS density equals #CLS/cm^2^.

Previously, the chemokine CCL2 has been implicated in the recruitment of blood monocytes into fat where the cells differentiate into macrophages contributing to the formation of CLS^13,14^. Given the observed increase in macrophages in the breast tissue of normal-weight women with high vs. low trunk fat, we evaluated the relationship between levels of trunk fat (trunk fat percent) and *CCL2* expression. As shown in Fig. 3a, the percentage of trunk fat positively correlated with *CCL2* expression (rho = 0.64, *P* = 9.4e−05). Notably, the level of *CCL2* expression also positively correlated with CD68 expression (rho = 0.47, *P* = 0.007) and the density of CLS (rho = 0.37, *P* = 0.04) (Fig. 3b, c). In addition to *CCL2*, complement is believed to be important for leukocyte recruitment into adipose tissue. Activation of the C3A receptor (C3AR), a component of the complement pathway, attracts inflammatory cells including monocytes into adipose tissue contributing to CLS formation^15^. Consistent with the increased macrophage population found in the breast tissue of normal BMI women with high vs. low levels of trunk fat, levels of *C3AR1* positively correlated with trunk fat percentage (rho = 0.56, *P* = 0.001; Fig. 3d). Levels of *C3AR1* expression also positively correlated with CD68 expression (rho = 0.79, *P* = 9.3e−07) and density of CLS (rho = 0.44, *P* = 0.01; Fig. 3e, f).

### Additional molecular changes correlate with the amount of trunk fat

**Fig. 4:**
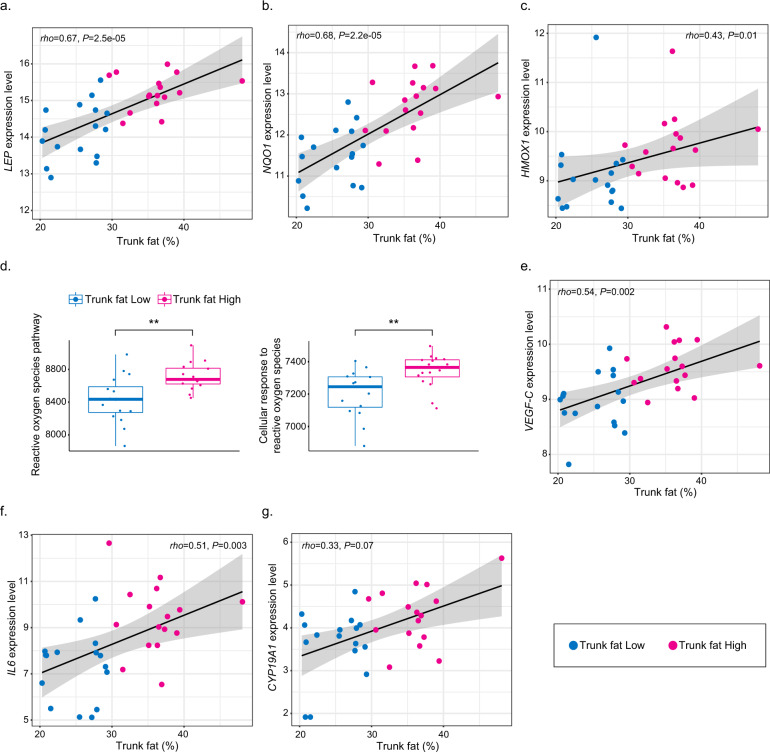
Levels of trunk fat correlate with the expression of genes implicated in the pathogenesis of breast cancer. **a** Correlation between trunk fat percent vs. *LEP* expression level. **b** Correlation between trunk fat percent vs. *NQO1* expression level. **c** Correlation between trunk fat percent vs. *HMOX1* expression level. **d** Pathway enrichment scores of reactive oxygen species pathway from Hallmark and cellular response to reactive oxygen species pathway from Gene Ontology. **P < 0.01. **e** Correlation between trunk fat percent and *VEGF-C* expression level. **f** Correlation between trunk fat percent and *IL6* expression level. **g** Correlation between trunk fat percent and *CYP19A1* expression level.

Obesity has been associated with changes in gene expression in normal breast tissue that are believed to contribute to the pathogenesis of breast cancer^16^. In contrast, little is known about the expression of select genes linked to carcinogenesis in the breast tissue of normal-weight women with high vs. low trunk fat. Hence, we evaluated the relationship between trunk fat percentage and the expression of several genes that potentially play a role in the pathogenesis of breast cancer. Some studies have suggested that leptin contributes to the pathogenesis of breast cancer^17^. Moreover, blood levels of leptin are elevated in normal-weight women with high levels of trunk fat and B-WATi^7,8^. Here we observed a strong correlation between trunk fat percentage and leptin expression (rho = 0.67, *P* = 2.5e−05; Fig. 4a). Excess body fat has been suggested to be associated with reduced oxygen levels in adipose tissue^18^. Tissue hypoxia is believed to stimulate the synthesis of reactive oxygen species which can, in turn, damage DNA potentially contributing to mutagenesis. Both *NQO1* and *HMOX1* are known to be induced by reactive oxygen species^19^. Interestingly, we observed a positive correlation between trunk fat percentage and the expression of both *NQO1* (rho = 0.68, *P* = 2.2e−05) and *HMOX1* (rho = 0.43, *P* = 0.01), which may reflect an increase in reactive oxygen species (Fig. 4b, c). In support of this potential mechanism, the reactive oxygen species pathway gene set from the Hallmark database and cellular response to reactive oxygen species from the Gene Ontology database were both elevated in association with high trunk fat (Fig. 4d). Obesity has also been reported to cause lymphatic dysfunction which could, in turn, impact the development and progression of breast cancer^20^. With this in mind, it is of considerable interest that levels of *VEGF-C*, which can promote the growth of lymphatic vessels, positively correlated with trunk fat percentage (rho = 0.54, *P* = 0.002; Fig. 4e). Interleukin (IL)-6, a pro-inflammatory cytokine, may also play a role in tumorigenesis. Systemic levels of IL-6 are elevated in association with obesity and B-WATi^21^. Here we demonstrate a positive correlation between trunk fat percentage and the expression of *IL6* (rho = 0.51, *P* = 0.003; Fig. 4f). We also found evidence suggesting that levels of *CYP19A1* which encodes aromatase, the rate-limiting enzyme for estrogen biosynthesis, were increased in association with high vs. low levels of trunk fat (log2FoldChange = 0.95; *P* = 0.01). A nearly significant positive correlation was found between trunk fat percentage and the expression of *CYP19A1* (rho = 0.33, *P* = 0.07; Fig. 4g).

## Discussion

Obesity is widely recognized to be a risk factor for numerous diseases, including solid and liquid tumors^2^. The fact that MONW was recently found to be associated with an increased risk of cancer suggests that the oncological consequences of excess body fat are greater than previously estimated in studies limited to the effects of obesity^4,8^. Although the association between high levels of trunk fat and elevated risk of postmenopausal breast cancer in MONW is now established^8^, there is a limited understanding of the underlying mechanisms.

In the current study, we have provided new insights into the molecular changes that occur in the breast tissue of normal BMI women with high vs. low levels of trunk fat. The fact that there were numerous DEGs is consistent with prior evidence that obesity is associated with significant changes in gene expression in the normal breast^16^. In contrast to the findings for high vs. low levels of trunk fat, we did not see a large difference in gene expression when relying on measurements of BMI. This finding underscores both the known limitations of BMI as a measurement of adiposity and the value of carrying out objective measures of body fat. Consistent with the previously established link between excess trunk fat in normal BMI women and B-WATi^5^, the immune score was elevated in the breast tissue of women with high compared with low trunk fat. The fact that both macrophages and dendritic cells were suggested to be increased in association with elevated trunk fat is consistent with the increased immune score. Future studies are warranted to determine whether the function of these immune cells is altered in association with excess trunk fat. It is possible, for example, that changes in both the number and function of these immune cells could contribute to the pathogenesis of breast cancer in MONW. Our results also provide insights into the mechanisms underlying the observed increase in macrophages in association with high trunk fat. The fact that we found elevated levels of both *CCL2* and *C3AR1* in association with high levels of trunk fat is likely to be important for explaining the link between MONW and B-WATi. In preclinical studies, both *CCL2* and activation of the complement pathway have been shown to impact WATi. CCL2 is a chemokine produced by adipocytes that is important for the recruitment of monocytes from blood into fat leading to the formation of CLS^13,14^. Levels of C3 complement are elevated in association with MONW^22^. C3 is upstream of the anaphylatoxin C3a, which binds to the C3a receptor, and thereby attracts inflammatory cells including monocytes into adipose tissue contributing to CLS formation^15^.

Several other findings are worth commenting on because of their potential link to breast carcinogenesis. Some studies have suggested a role for leptin in explaining the link between obesity and increased risk of breast cancer^17^. Leptin is produced by adipocytes and is known to be elevated in the blood of normal BMI postmenopausal women with increased trunk fat^8^. The fact that we found leptin levels to be increased in the breast tissue of normal BMI women with high vs. low trunk fat is consistent with the increase in breast adipocyte size that was observed. Whether this local increase in leptin contributes to the elevated risk of breast cancer is uncertain but warrants further consideration. Excess body fat is associated with hypoxia in adipose tissue which can cause an increase in reactive oxygen species^18^. *NQO1* and *HMOX1* are Nrf-2-dependent genes that are induced by reactive oxygen species^19^, a cause of oxidative DNA damage. Here we demonstrated that levels of both *NQO1* and *HMOX1* were elevated in association in high vs. low trunk fat. Moreover, pathway analysis suggested an increase in reactive oxygen species in association with high trunk fat. Taken together, it is possible that excess trunk fat is associated with an increased risk of breast cancer because the chronic elevation of reactive oxygen species may increase the likelihood of mutagenesis. The observed elevation of *IL6* in association with high vs. low trunk fat is consistent with a pro-inflammatory state and prior evidence from reduction mammoplasty specimens that obesity led to enrichment for a pathway involving *IL6*^16^. Local increases in *IL6* could impact the self-renewal of cancer stem cells or potentially the growth of breast cancer cells^23^. Elevated levels of *CYP19A1* which encodes aromatase have previously been reported in association with excess body fat including in normal BMI women^5,7^. This increase could help to explain the elevated risk of estrogen-dependent breast cancer in normal BMI postmenopausal women^8^^,24^.

This study has both strengths and limitations. A major strength is the use of RNA-seq to describe global changes in the breast transcriptome in association with high vs. low trunk fat in normal BMI women. This strategy has provided significant new mechanistic insights into the link between MONW and elevated risk of breast cancer. The fact that we had objective DXA-derived measurements of trunk fat and didn’t rely on BMI was a major strength of the study. At the same time, there were limitations including both the small sample size and the number of postmenopausal women who were enrolled. In the future, it would be very useful to both expand the sample size and to compare the effects of high vs. low trunk fat on gene expression in postmenopausal vs. premenopausal women. The nontumorous tissue that we evaluated came from patients either at elevated risk for breast cancer or with breast cancer. Although in cancer patients, we utilized tissue from an uninvolved quadrant of the breast, it is possible that the results could be different in normal women. Hence, in the future, it would be worthwhile to carry out a validation study using normal breast tissue from a cohort of cancer-free patients. Our findings are also limited because we are not able to delineate individual contribution to the variation in gene expression from all variables of interest including trunk fat, age, BMI, CLS status, and adipocyte size due to the limited sample size and the correlations between these variables. Our separate analyses provide evidence of the existence of trunk fat-related gene expression signatures. Nonetheless, such findings should be validated in larger studies. Finally, bulk RNA-seq allowed us to identify important differences between normal BMI women with high vs. low trunk fat, but we were unable to determine whether the observed differences in gene expression reflect changes in transcription, cellular composition of the tissue or both. The current findings provide a strong rationale for future studies utilizing single-cell RNA-seq.

In summary, we demonstrate that there are significant changes in gene expression in the breast tissue of normal BMI women with high vs. low levels of trunk fat that reflect alterations in the complement pathway, cytokines and inflammatory response, cytokine–cytokine receptor interaction, and reactive oxygen species. Changes in immune cells and the expression of numerous genes linked to the pathogenesis of cancer were found that may help to explain why MONW is associated with an increased risk of postmenopausal breast cancer.

## Methods

### Study population and clinical data collection

The subjects in this study of normal BMI women represent a subgroup of a previously reported cohort study that included measurements of body composition, B-WATi, and breast adipocyte diameter in women of all sizes^5^. In the previous study, the breast transcriptome was not evaluated. Informed consent was provided by women who were scheduled to undergo mastectomy for breast cancer treatment or risk reduction at Memorial Sloan Kettering Cancer Center (MSKCC). Institutional Review Board approval was obtained from MSKCC (IRB Protocol Number 15-235) and Weill Cornell Medicine (IRB Protocol Number 1511016737) (New York, NY) to conduct this study. Trunk fat was measured by DXA using a Lunar Prodigy multiple detector fan-beam densitometer (GE Healthcare) before surgery. Following calibration, single-beam, whole-body scanning was performed in the supine position. Weight and height were recorded before surgery and used to calculate BMI. A standard definition was used to define normal BMI (18.5–<25 kg/m^2^). Clinicopathological data (age, menopause status, race) were extracted from the electronic medical record. Menopause status was categorized as either postmenopausal or premenopausal based on National Comprehensive Cancer Network criteria^25^.

### Biospecimen acquisition

Breast WAT specimens were obtained prospectively under a standard tissue acquisition protocol. In specimens that contained tumors, tissue from an uninvolved quadrant of the breast was used for research purposes. For each subject, paraffin blocks were prepared from breast WAT not involved by a tumor on the day of surgery. Frozen samples were stored in the presence RNAlater (Ambion).

### Detection and measurement of B-WATi

The absence or presence of B-WATi was determined by histologic assessment as described before^26,27^. Briefly, B-WATi was detected by the presence of CLS, which represents a dead or dying adipocyte surrounded by CD68-positive macrophages^28^. Five formalin-fixed paraffin-embedded (FFPE) blocks were prepared from each mastectomy specimen and one section per FFPE block (approximately 2 cm in diameter, 5 µm thick) was generated such that five sections were stained for CD68, a macrophage marker (mouse monoclonal KP1 antibody; Dako). Light microscopy was used to examine immunostained tissue sections to detect the presence or absence of CLS and record the number of CLS per slide. Digital photographs were generated and the WAT area was measured with the Image J Software (NIH, Bethesda, MD). The number of CLS per square centimeter of WAT (#CLS/cm^2^) also referred to as CLS density was calculated to determine the severity of B-WATi.

### Adipocyte measurement

Two hematoxylin & eosin-stained sections per case were prepared from FFPE breast tissue in order to measure adipocyte diameters as previously described^5^. Adipocyte diameter is expressed in microns.

### RNA isolation and sequencing

Qiagen’s RNeasy minikit was used to isolate total RNA from non-tumorous breast tissue. Then, using the Illumina TrueSeq Stranded Total RNA Library preparation protocol with rRNA depletion, the libraries were constructed. RNA-seq was carried out with paired-end 51 bp using the Illumina HiSeq4000 platform (Weill Cornell Medicine). Raw sequenced reads were pseudoaligned to the human reference genome (UCSC hg19) using Kallisto^29^. Then, the transcript abundance was quantified to obtain raw counts.

### DEG analysis

Raw counts acquired from Kallisto were used to characterize DEGs and DESeq2 R package was used^30^. The genes with low expression values were filtered out (baseMean ≤ 15) first. Then, genes with adjusted *P* value < 0.05 and |Log_2_FoldChange| > 0.6 were defined as DEGs. Volcano plots were generated to visually display the most biologically significant genes using statistical significance vs. fold-change. Two vertical dashed lines correspond to −0.6 and 0.6, which represent one of the cutoffs for the DEG. The horizontal dashed line corresponds to *P* adjusted value of 0.05, which is another cutoff for the DEG.

### Pathway enrichment analysis

To determine the molecular function of DEGs in various biological processes, KEGG^31,32^ and BioCarta^31,33^ gene sets from the Molecular signature database were used.

### Immune cell population analyses

To determine whether the overall immune cell population in breast WAT changed in association with percent trunk fat, Estimate R package was used to generate an immune score^11^. Then, specific immune cell-type changes were determined using a webtool, xCell (https://xcell.ucsf.edu/)^34^. To confirm findings from xCell, immune cell expression markers from the study of Danaher et al. were used^12^.

### Pathway score analysis

To determine whether a given pathway is coordinately upregulated or downregulated in a sample set, single-sample GSEA (ssGSEA) was used. ssGSEA calculates an enrichment score for each gene set and the score represents the activity level of a biological process. The analysis was carried out on the public server (https://www.genepattern.org/modules/docs/ssGSEAProjection/4). Gene sets from Hallmark and Gene Ontology databases were used^31,35–37^.

### Statistics

Patient characteristics were summarized in terms of counts and proportions for categorical variables and median and interquartile range for continuous variables. Fisher’s exact test was used to examine the difference in the distribution of a categorical variable between two independent groups. Wilcoxon rank-sum test was used to examine the difference of a continuous variable between two independent groups. Correlation between two continuous variables was examined using Spearman’s method. For all analyses, statistical significance was set at two-tailed *P* < 0.05. All statistical analyses were conducted using the R software (R Foundation for Statistical Computing, Vienna, Austria).

### Reporting summary

Further information on research design is available in the Nature Research Reporting Summary linked to this article.

## Supplementary information

Supplementary Information

Reporting Summary

## Data Availability

To protect patient privacy, the data that support this study are not publically available. Data will be provided to researchers who have received authorization from the MSKCC Institutional Review Board. To request access to data, please contact N.M.I., email address: iyengarn@mskcc.org with requests. All RNA-sequencing data have been deposited at the European Genome-phenome Archive (EGA), which is hosted by the EBI and the CRG, under accession number EGAS00001005138.
