# Supplementary material for: Increased trunk fat is associated with altered gene expression in breast tissue of normal weight women
Source: NPJ Breast Cancer. 2022 Jan 27;8:15. doi: 10.1038/s41523-021-00369-8 (PMC8795267; doi:10.1038/s41523-021-00369-8)
Supplement: Supplementary file 1 — Supplementary Information [file 41523_2021_369_MOESM1_ESM.pdf]

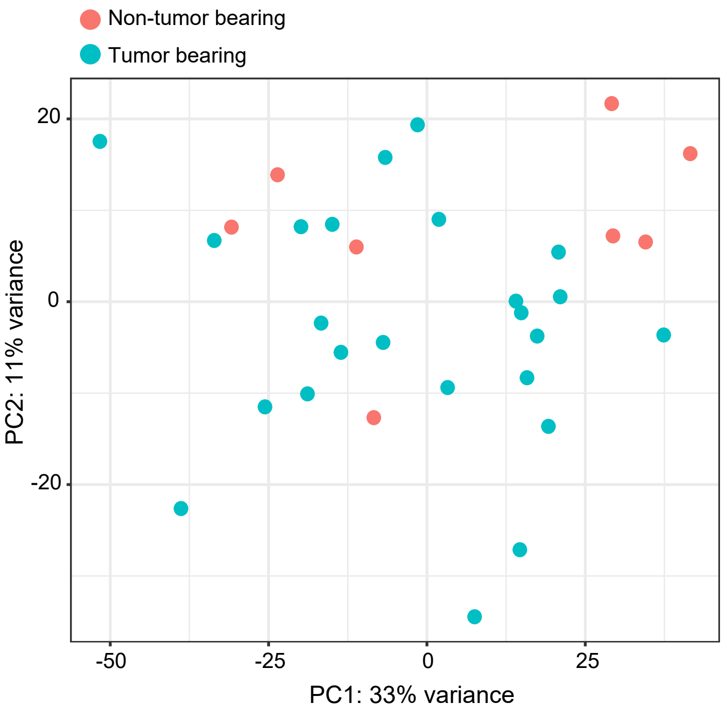

Supplementary Figure 1. Principal component analysis demonstrates that the presence of tumor had no effect on the transcriptome of non-tumorous breast tissue from an uninvolved quadrant of the breast. Non-tumor bearing and tumor bearing breast samples are well interspersed without distinctive clusters formed based on tumor status.

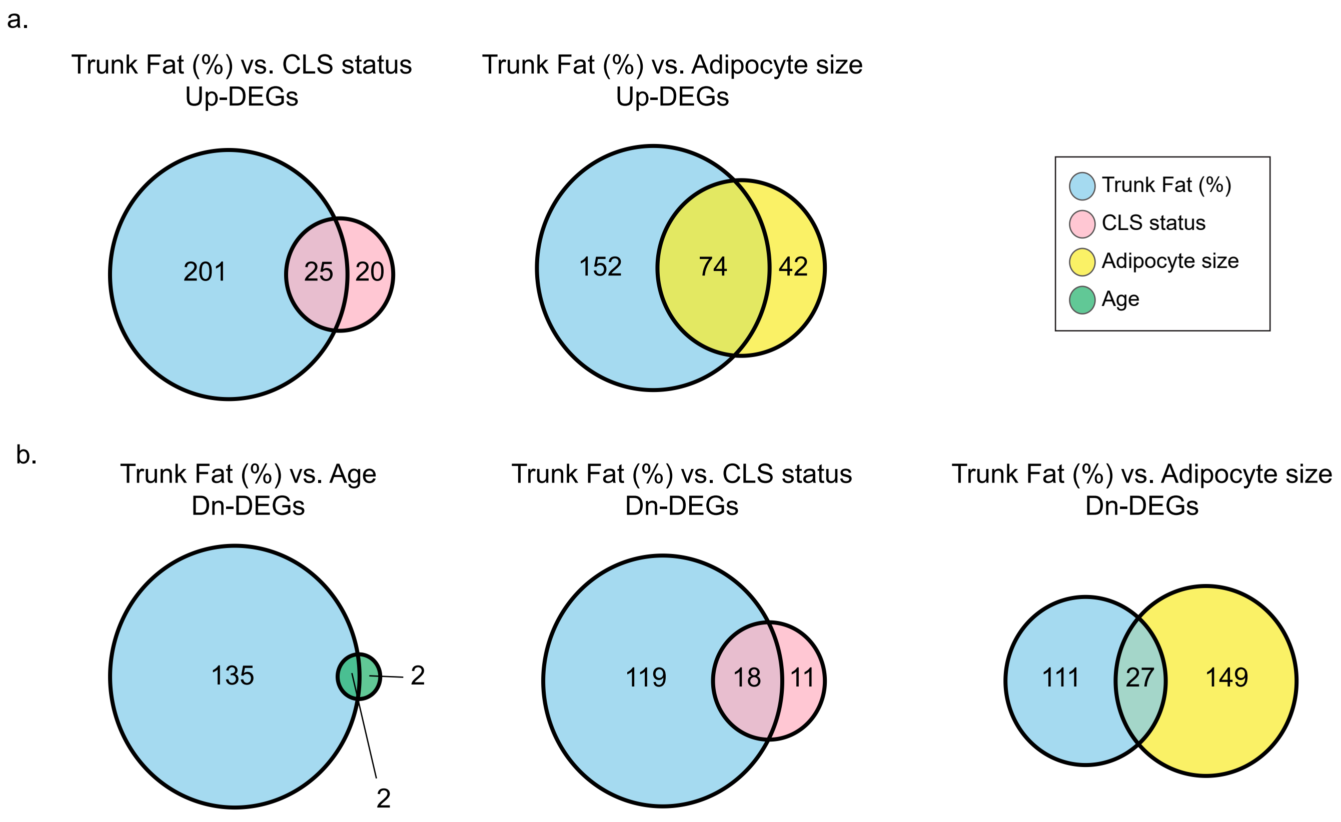

Supplementary Figure 2. Venn diagrams describing the number of overlapping DEGs. a. Up-regulated DEGs. b. Down-regulated DEGs.

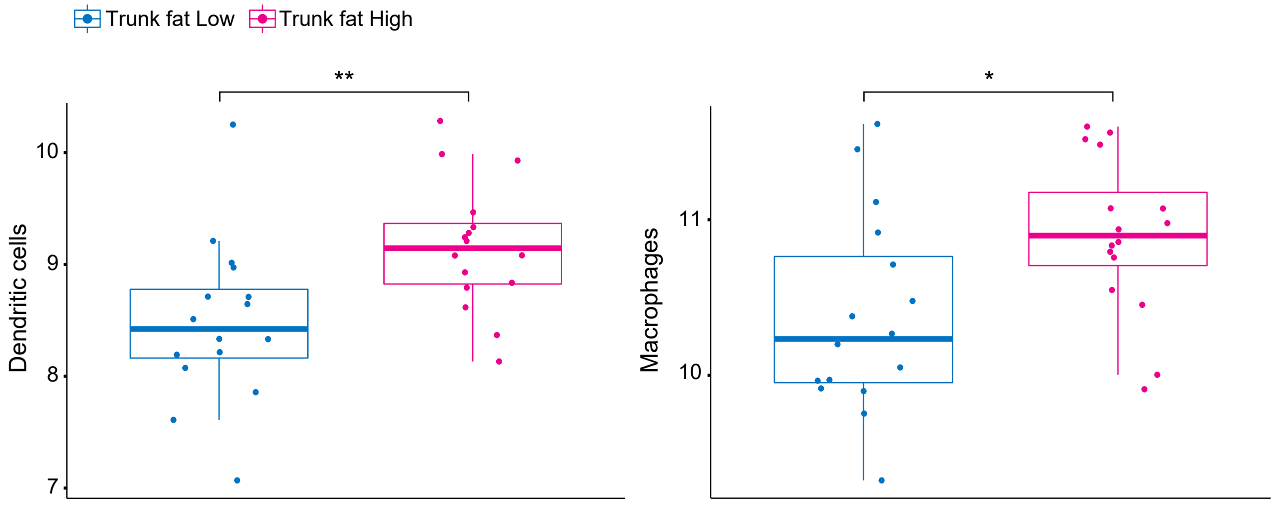

Supplementary Figure 3. Increased populations of dendritic cells and macrophages were found in association with high vs. low trunk fat in normal BMI women. This analysis was carried out based on the gene expression signatures of Danaher et al. *P<0.05; **P<0.01

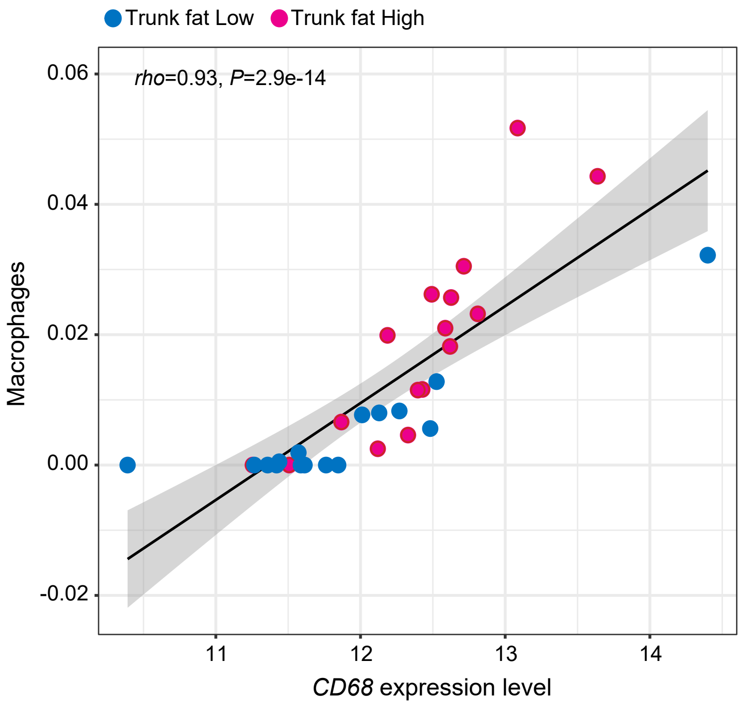

Supplementary Figure 4. Levels of *CD68*, a macrophage marker, correlate with the estimated macrophage population in breast white adipose tissue as determined by xCell.

Supplementary Table 1. Differentially expressed genes in trunk fat high vs. low. P.adj<0.05, |Log2FoldChange|>=0.6

Gene	DEG	Log2FoldChange	P.adj
IL1RN	UP	2.03	2.01E-08
CSF3	UP	3.31	1.82E-05
UCHL1	UP	2.01	1.82E-05
HSPB8	UP	1.04	7.30E-05
HCST	UP	1.30	9.68E-05
LINC01852	UP	0.88	9.68E-05
PLAC9	UP	1.36	9.68E-05
SIGLEC7	UP	1.43	0.0002
ARHGDIG	UP	2.07	0.0002
TYROBP	UP	1.26	0.0002
DPT	UP	1.27	0.0002
CD83	UP	1.11	0.0002
LOC101929398	UP	1.95	0.0002
NQO1	UP	1.23	0.0003
TREM1	UP	1.43	0.0003
SYNC	UP	1.07	0.0003
ANKDD1A	UP	1.17	0.0004
NLRP3	UP	1.10	0.0005
TIMP1	UP	0.87	0.0006
CRYBB1	UP	1.13	0.0007
CRYAB	UP	1.32	0.0007
HSD11B1	UP	1.14	0.0007
EPDR1	UP	0.83	0.0008
SPI1	UP	0.78	0.0008
IFITM3	UP	0.77	0.0011
CDA	UP	1.13	0.0013
DOK2	UP	1.03	0.0013
SRPX2	UP	0.90	0.0014
LOC107987423	UP	1.39	0.0016
GNA15	UP	0.92	0.0017
ARPC1B	UP	0.65	0.0017
FCGR2A	UP	0.79	0.0018
CCL3	UP	1.58	0.0019
VMO1	UP	1.27	0.0019
HAS1	UP	2.26	0.0019
CPVL	UP	0.70	0.0020
FERMT3	UP	0.76	0.0020
WAS	UP	0.71	0.0020
CCND2	UP	0.70	0.0020
LEP	UP	0.98	0.0020
IL6	UP	2.18	0.0021
LOC105377918	UP	1.35	0.0021

CLEC2L	UP	1.77	0.0022
LINC00968	UP	1.65	0.0023
SLC14A2-AS1	UP	1.53	0.0025
FPR3	UP	0.88	0.0025
MYMX	UP	1.19	0.0025
C11orf96	UP	1.15	0.0026
LST1	UP	1.01	0.0026
C1QA	UP	1.09	0.0027
LOC102724850	UP	1.21	0.0027
IFITM1	UP	0.61	0.0027
EMP3	UP	0.74	0.0027
SAA1	UP	1.55	0.0030
MSR1	UP	0.88	0.0031
TNMD	UP	0.98	0.0032
C1QB	UP	1.02	0.0034
JUND	UP	0.75	0.0035
VLDLR-AS1	UP	1.95	0.0038
CD300C	UP	0.95	0.0042
MYL9	UP	0.89	0.0042
HMOX1	UP	0.93	0.0043
CCN5	UP	0.85	0.0045
UBE2D4	UP	0.60	0.0045
PLA1A	UP	1.01	0.0048
BAG3	UP	0.82	0.0049
CCL18	UP	1.88	0.0051
CXCL9	UP	1.30	0.0051
METRNL	UP	0.73	0.0051
PLAAT1	UP	1.03	0.0051
IFITM2	UP	0.65	0.0052
SH2D4A	UP	0.65	0.0052
ACP5	UP	1.13	0.0053
CYP4Z2P	UP	1.26	0.0055
P2RX6	UP	1.68	0.0059
C5orf64	UP	1.04	0.0067
PARD6A	UP	0.74	0.0067
SLC2A3	UP	1.03	0.0067
VENTX	UP	0.84	0.0067
GZMK	UP	1.23	0.0069
C1QC	UP	1.01	0.0070
CD151	UP	0.77	0.0076
LCP1	UP	0.60	0.0081
MMP19	UP	0.81	0.0081
DUSP14	UP	0.63	0.0082
SH3BGRL3	UP	0.68	0.0082
LINC00294	UP	0.72	0.0082
LGALS9	UP	0.60	0.0087
HS3ST2	UP	1.34	0.0091

HTRA1	UP	0.68	0.0093
LINC00857	UP	0.67	0.0096
CCL2	UP	1.28	0.0100
RND1	UP	1.41	0.0101
BTK	UP	0.67	0.0104
IL18	UP	0.75	0.0106
TAGLN	UP	0.70	0.0108
FCGR1B	UP	1.34	0.0109
CCDC3	UP	1.40	0.0111
RHOXF1	UP	1.08	0.0112
ADAMTS4	UP	1.32	0.0118
CD86	UP	0.83	0.0120
S100A10	UP	0.74	0.0124
TMEM54	UP	0.80	0.0140
BOLA2	UP	0.66	0.0143
LOC105372270	UP	1.14	0.0154
SELE	UP	2.09	0.0154
LOC101927243	UP	1.31	0.0157
GPSM3	UP	0.64	0.0161
GASAL1	UP	0.68	0.0174
IFI27L2	UP	0.68	0.0175
SNORD140	UP	2.61	0.0175
CH25H	UP	1.30	0.0177
ELN	UP	0.70	0.0177
TRDN	UP	1.62	0.0177
AFF2	UP	1.32	0.0181
EPB42	UP	1.28	0.0181
ITGB1BP1	UP	0.66	0.0181
LOC105374413	UP	0.67	0.0181
TMIGD3	UP	0.86	0.0184
URAD	UP	2.75	0.0184
BHMT2	UP	0.76	0.0191
PEMT	UP	0.79	0.0195
SCIN	UP	1.23	0.0195
SAA2	UP	1.49	0.0196
CASP4LP	UP	0.63	0.0199
MS4A14	UP	1.04	0.0206
FBLN1	UP	0.62	0.0210
PLTP	UP	0.68	0.0212
SLC15A3	UP	0.62	0.0212
LOC105369488	UP	1.40	0.0213
PIM1	UP	1.01	0.0213
RPP25	UP	0.61	0.0213
ULK4	UP	0.64	0.0213
LOC112267855	UP	0.93	0.0213
CD14	UP	0.70	0.0217
JAML	UP	0.83	0.0217

CD1C	UP	1.02	0.0220
VEGFC	UP	0.62	0.0220
C1QTNF5	UP	0.75	0.0226
GPRC5A	UP	0.99	0.0226
C15orf65	UP	0.63	0.0234
LHCGR	UP	1.35	0.0236
PYCARD	UP	0.68	0.0236
DNLZ	UP	0.93	0.0238
PIR	UP	0.65	0.0242
THEMIS2	UP	0.73	0.0242
TMEM160	UP	0.75	0.0242
FCGR1A	UP	0.97	0.0242
FGF18	UP	1.12	0.0242
KLHL30	UP	1.17	0.0242
MXRA7	UP	0.62	0.0243
CTSS	UP	0.61	0.0247
CST3	UP	0.77	0.0248
ISG15	UP	0.86	0.0256
ADAP1	UP	0.71	0.0265
CD52	UP	1.20	0.0266
TNFSF14	UP	0.73	0.0276
AIF1	UP	0.83	0.0277
CLEC4G	UP	1.28	0.0277
LOC389705	UP	0.66	0.0285
CHIT1	UP	1.85	0.0286
RAB32	UP	0.71	0.0290
MYZAP	UP	0.72	0.0292
PMEPA1	UP	0.76	0.0292
C2CD4B	UP	1.52	0.0297
RNF122	UP	0.96	0.0297
BCL2A1	UP	1.08	0.0298
FOLR2	UP	0.85	0.0298
NPL	UP	0.70	0.0302
ADCYAP1	UP	1.75	0.0307
ZNF541	UP	1.02	0.0307
LOC105378687	UP	0.63	0.0315
IL17D	UP	0.67	0.0320
LINC01260	UP	1.00	0.0322
SULT1A2	UP	1.13	0.0333
ACOT7	UP	0.61	0.0336
NCF2	UP	0.62	0.0339
LOC101929638	UP	0.88	0.0342
HLA-DRA	UP	0.64	0.0345
RENBP	UP	0.72	0.0345
LINC01402	UP	0.85	0.0347
RGS16	UP	0.78	0.0347
TPSD1	UP	1.56	0.0355

RASGRP4	UP	0.65	0.0357
VLDLR	UP	0.83	0.0368
ITGB2	UP	0.67	0.0369
SLC51A	UP	0.69	0.0373
C19orf38	UP	0.93	0.0373
PROK1	UP	1.56	0.0375
NPR3	UP	1.12	0.0377
NRROS	UP	0.61	0.0379
C1orf198	UP	0.77	0.0390
PISD	UP	0.63	0.0390
HLA-DPB1	UP	0.72	0.0390
TLCD2	UP	0.61	0.0399
CD300E	UP	0.84	0.0407
GRB14	UP	0.82	0.0407
RAB44	UP	0.76	0.0407
EBI3	UP	0.79	0.0417
KLHDC7A	UP	1.69	0.0428
CD53	UP	0.67	0.0429
GPR183	UP	0.75	0.0429
PDLIM2	UP	0.61	0.0429
SNHG26	UP	0.89	0.0432
NCF1	UP	0.68	0.0442
ZNF219	UP	0.73	0.0442
CES1	UP	0.90	0.0443
MIEN1	UP	0.61	0.0443
BCYRN1	UP	1.08	0.0446
HK3	UP	0.81	0.0452
FAM180B	UP	0.93	0.0464
LOC105375707	UP	0.78	0.0467
MIR22HG	UP	0.61	0.0467
IGSF6	UP	0.71	0.0468
SOCS3	UP	1.11	0.0471
C1orf122	UP	0.76	0.0472
AKR1C1	UP	0.67	0.0473
IER3-AS1	UP	1.60	0.0473
CFP	UP	0.67	0.0488
FHL1	UP	0.69	0.0488
PI16	UP	0.93	0.0488
IGFBP6	UP	0.63	0.0489
C3AR1	UP	0.71	0.0492
HSPB2	UP	0.69	0.0496
CFD	UP	0.72	0.0497
NTNG1	UP	0.97	0.0498
CSN2	DN	-9.40	0.0000
LALBA	DN	-7.74	0.0000
LOC102723566	DN	-0.92	0.0000
BTN1A1	DN	-4.23	0.0000

LOC105375012	DN	-3.35	0.0000
CSN3	DN	-6.62	0.0000
XDH	DN	-2.49	0.0001
BRIP1	DN	-0.98	0.0001
CHIA	DN	-3.68	0.0001
SLC28A3	DN	-3.20	0.0007
C6	DN	-1.71	0.0007
LOC107985388	DN	-0.89	0.0020
PKP2	DN	-1.05	0.0021
MGAM2	DN	-2.31	0.0021
ALK	DN	-1.27	0.0022
PI3	DN	-2.31	0.0025
SEMA4G	DN	-0.60	0.0036
SLC16A10	DN	-0.64	0.0037
SLCO1C1	DN	-1.05	0.0045
TTC36	DN	-1.34	0.0048
LOC107987152	DN	-0.96	0.0050
MAMDC4	DN	-1.50	0.0052
MEF2B	DN	-1.68	0.0052
PEX5L	DN	-0.99	0.0054
MOGAT1	DN	-1.29	0.0057
LTF	DN	-2.00	0.0059
GFAP	DN	-1.04	0.0067
LOC105378577	DN	-2.00	0.0070
ABCG8	DN	-0.71	0.0071
MUC5B	DN	-2.13	0.0082
GCK	DN	-1.28	0.0091
TOX3	DN	-1.53	0.0091
AMN	DN	-1.67	0.0098
OBSCN	DN	-0.93	0.0098
CLCA4	DN	-2.40	0.0099
FGF7P3	DN	-0.81	0.0114
LINC02076	DN	-0.93	0.0115
ATP2B2	DN	-1.43	0.0116
KIF19	DN	-1.27	0.0124
TMEM9B-AS1	DN	-0.76	0.0125
SSPOP	DN	-0.93	0.0126
LOC101927300	DN	-0.63	0.0134
PKIA	DN	-0.66	0.0140
ZNF334	DN	-0.93	0.0140
SLC16A8	DN	-0.92	0.0156
CECR2	DN	-0.94	0.0157
LTB4R2	DN	-0.62	0.0159
CA3	DN	-1.51	0.0161
CSPG5	DN	-0.88	0.0169
MYBL2	DN	-0.92	0.0169
BBOX1	DN	-1.59	0.0172

LOC102723648	DN	-0.82	0.0173
GLYCTK	DN	-1.12	0.0174
LOC105378561	DN	-0.73	0.0177
LOC107984836	DN	-0.91	0.0191
C19orf57	DN	-0.78	0.0192
LINC01235	DN	-1.91	0.0195
LOC101927401	DN	-1.19	0.0199
ALG1L	DN	-0.61	0.0202
TBATA	DN	-1.98	0.0208
LOC105373903	DN	-1.09	0.0209
COL27A1	DN	-0.92	0.0213
CTSV	DN	-1.26	0.0213
LOC105372431	DN	-0.90	0.0213
LPIN1	DN	-0.77	0.0213
TIAM2	DN	-0.95	0.0213
SLC34A2	DN	-1.97	0.0218
DNHD1	DN	-0.63	0.0220
PLEKHS1	DN	-1.87	0.0220
PRRT4	DN	-1.09	0.0220
CDT1	DN	-1.24	0.0222
PEG10	DN	-1.13	0.0236
RAB26	DN	-1.13	0.0246
RP1L1	DN	-1.07	0.0246
SHROOM2	DN	-0.86	0.0246
ANKRD20A8P	DN	-1.32	0.0247
SLC9A3	DN	-0.61	0.0255
ABCG5	DN	-0.74	0.0259
LOC107986785	DN	-0.85	0.0259
LOC727978	DN	-0.93	0.0259
NRAD1	DN	-1.46	0.0259
KIF20A	DN	-1.23	0.0261
LOC105374473	DN	-1.01	0.0266
LOC105369201	DN	-1.33	0.0269
FAM161A	DN	-0.64	0.0271
PCP4L1	DN	-1.04	0.0279
EPCAM-DT	DN	-1.04	0.0283
B3GAT1	DN	-1.42	0.0287
KLHL32	DN	-0.85	0.0298
PDZD7	DN	-0.98	0.0309
GLUD1P3	DN	-0.63	0.0314
AZGP1P1	DN	-1.23	0.0317
LOC101927088	DN	-0.83	0.0317
ANKRD24	DN	-0.68	0.0322
MUC16	DN	-1.45	0.0328
FABP7	DN	-2.19	0.0328
FASN	DN	-1.19	0.0329
EYA1	DN	-0.88	0.0333

LOC105373027	DN	-1.41	0.0333
KRT80	DN	-1.79	0.0337
MRPL23-AS1	DN	-0.99	0.0341
PTCHD1	DN	-1.49	0.0345
TCN1	DN	-1.90	0.0355
ANKK1	DN	-0.66	0.0365
PWARSN	DN	-0.66	0.0368
GOLGA6L9	DN	-0.81	0.0369
PPP1R14C	DN	-1.47	0.0372
NPC1L1	DN	-1.01	0.0382
KCNS1	DN	-1.85	0.0390
KCTD14	DN	-1.34	0.0390
LOC107985939	DN	-0.74	0.0390
LRIG1	DN	-0.71	0.0390
ANTXRL	DN	-1.66	0.0394
SCNN1G	DN	-1.43	0.0395
CEL	DN	-1.12	0.0402
RIMBP2	DN	-0.96	0.0421
SMIM17	DN	-1.84	0.0428
HELLS	DN	-0.94	0.0429
PRR7	DN	-1.09	0.0429
SPTB	DN	-1.01	0.0429
LOC101927751	DN	-1.11	0.0434
CSN1S2AP	DN	-9.53	0.0442
TSPY26P	DN	-0.81	0.0442
ESPL1	DN	-0.98	0.0443
MAGED4	DN	-0.81	0.0452
LOC105371692	DN	-0.75	0.0454
LOC105371505	DN	-0.89	0.0461
LOC112268187	DN	-1.34	0.0464
LINC00680-GUSBP4	DN	-0.85	0.0468
ADIPOQ-AS1	DN	-1.25	0.0473
LOC112268021	DN	-0.82	0.0473
ACSM2B	DN	-0.84	0.0479
TROAP	DN	-1.07	0.0485
INTS4P2	DN	-1.81	0.0488
RASD2	DN	-0.93	0.0491
UHRF1	DN	-0.94	0.0494
FAM166B	DN	-0.86	0.0496

Supplementary Table 2. Differentially expressed genes for age>= 45 vs. age<45 years. P.adj<0.05, |Log2FoldChange|>=0.6

Gene	DEG	Overlapped with High vs. Low Trunk Fat	Log2FoldChange	P.adj
DHRS2	UP	No overlap	2.03	0.029804
CSN2	DN	Overlapped	-6.92	0.029804
LALBA	DN	Overlapped	-5.67	0.029804
LINC00639	DN	No overlap	-0.79	0.029804

Supplementary Table 3. Differentially expressed genes in post- vs. pretmenopausal women. P.adj<0.05, |Log2FoldChange|>=0.6

Gene	DEG	Overlapped with High vs. Low Trunk Fat	Log2FoldChange	P.adj
LOC107984247	UP	No overlap	7.69	0.005367
LOC105372884	UP	No overlap	0.82	0.022463
RCAN2	UP	No overlap	1.26	0.035198
SLC16A9	DN	No overlap	-2.61	0.000003
TFF1	DN	No overlap	-5.25	0.001846
GREB1	DN	No overlap	-1.77	0.005367
LOC105379521	DN	No overlap	-2.06	0.005367
SCGB3A1	DN	No overlap	-4.48	0.006377
GLRA3	DN	No overlap	-3.04	0.018312
LINC01139	DN	No overlap	-3.03	0.033351
LINC01087	DN	No overlap	-3.64	0.041224
TUBA3D	DN	No overlap	-2.09	0.041224

Supplementary Table 4. Differentially expressed genes for CLS(+) vs. CLS(-). P.adj<0.05, |Log2FoldChange|>=0.6

Gene	DEG	Overlapped with High vs. Low Trunk Fat	Log2FoldChange	P.adj
UPK1A	UP	No overlap	2.21	0.000251294
ACP5	UP	Overlapped	1.37	0.000727725
CCL18	UP	Overlapped	2.24	0.001355663
RPP25	UP	Overlapped	0.77	0.007356342
VLDLR-AS1	UP	Overlapped	2.14	0.007922787
CCN5	UP	Overlapped	0.94	0.008988897
DPT	UP	Overlapped	1.22	0.008988897
IQCD	UP	No overlap	0.66	0.008988897
CHIT1	UP	Overlapped	2.34	0.009641139
TMIGD3	UP	Overlapped	1.01	0.016480607
ATP6V0D2	UP	No overlap	1.35	0.019248135
LINC02227	UP	No overlap	1.54	0.020926916
SNORD140	UP	Overlapped	2.95	0.020926916
LL22NC03-104C7.1	UP	No overlap	0.85	0.02124364
LOC105374413	UP	Overlapped	0.76	0.024222978
HS3ST2	UP	Overlapped	1.44	0.024900316
IL18	UP	Overlapped	0.81	0.027290226
ITGB1BP1	UP	Overlapped	0.75	0.027590712
SRPX2	UP	Overlapped	0.88	0.027590712
GFRAL	UP	No overlap	1.07	0.028219215
PLA2G7	UP	No overlap	1.31	0.031911888
ZBED5-AS1	UP	No overlap	0.67	0.032686129
LOC105374657	UP	No overlap	2.04	0.034716876
PRDX4	UP	No overlap	0.65	0.034716876
TXN	UP	No overlap	0.64	0.034716876
LINC01852	UP	Overlapped	0.76	0.034942895
BOLA2	UP	Overlapped	0.71	0.035130342
CASP4LP	UP	Overlapped	0.70	0.037165709
DOK2	UP	Overlapped	0.95	0.037165709
LOC101927243	UP	Overlapped	1.37	0.039003386
TNMD	UP	Overlapped	0.95	0.039003386
GGTA1P	UP	No overlap	0.64	0.039086782
RAB32	UP	Overlapped	0.81	0.039086782
UCHL1	UP	Overlapped	1.59	0.040036356
LOC105373334	UP	No overlap	0.92	0.046658069
MSTO1	UP	No overlap	0.71	0.046658069
FTH1	UP	No overlap	0.73	0.047321604
FTH1P3	UP	No overlap	0.85	0.047321604
HSD11B1-AS1	UP	No overlap	0.97	0.047321604
PLAAT1	UP	Overlapped	1.00	0.047321604
C15orf65	UP	Overlapped	0.68	0.048176156

CCDC13	UP	No overlap	0.68	0.049049103
GNA15	UP	Overlapped	0.84	0.049049103
NPC2	UP	No overlap	0.63	0.049049103
LOC101929645	UP	No overlap	0.63	0.049268263
CSN1S2AP	DN	Overlapped	-22.87	1.31E-08
LALBA	DN	Overlapped	-7.76	0.000105711
CSN2	DN	Overlapped	-8.79	0.000251294
PIGR	DN	No overlap	-3.24	0.007922787
BTN1A1	DN	Overlapped	-3.91	0.00858592
CSN3	DN	Overlapped	-6.06	0.019248135
TBATA	DN	Overlapped	-2.48	0.020569288
SHISA6	DN	No overlap	-0.95	0.024222978
LPIN1	DN	Overlapped	-0.92	0.026478841
MMS22L	DN	No overlap	-0.67	0.026478841
CDCA7	DN	No overlap	-1.37	0.032686129
MRPL23-AS1	DN	Overlapped	-1.26	0.033600066
LOC105371505	DN	Overlapped	-1.16	0.033863564
APOL4	DN	No overlap	-1.43	0.034716876
LOC105373903	DN	Overlapped	-1.29	0.034716876
PRDM1	DN	No overlap	-0.74	0.034716876
SLCO1C1	DN	Overlapped	-1.08	0.034817576
LOC102723566	DN	Overlapped	-0.75	0.038639229
MUC5B	DN	Overlapped	-2.27	0.038639229
ZNF334	DN	Overlapped	-1.01	0.039035293
CHRNA7	DN	No overlap	-1.30	0.039086782
CTH	DN	No overlap	-0.73	0.039086782
LINC02656	DN	No overlap	-0.89	0.039342033
ANKRD20A8P	DN	Overlapped	-1.53	0.041604506
BMP3	DN	No overlap	-1.92	0.041604506
GPR137C	DN	No overlap	-0.83	0.041604506
C6	DN	Overlapped	-1.53	0.047367673
PRRT4	DN	Overlapped	-1.20	0.049049103
MAMDC4	DN	Overlapped	-1.48	0.049268263

Supplementary Table 5. Differentially expressed genes in large vs. small adipocytes. P.adj<0.05, |Log2FoldChange|>=0.6

Gene	DEG	Overlapped with High vs. Low Trunk Fat	Log2FoldChange	P.adj
EPB42	UP	Overlapped	1.75	3.93E-05
LOC101929398	UP	Overlapped	2.00	0.00048954
LINC00968	UP	Overlapped	1.85	0.000603474
SCIN	UP	Overlapped	1.55	0.001156601
UCHL1	UP	Overlapped	1.86	0.001156601
CCDC3	UP	Overlapped	1.67	0.001211335
SLC14A2-AS1	UP	Overlapped	1.67	0.001211335
PLA1A	UP	Overlapped	1.12	0.00132494
ULK4	UP	Overlapped	0.78	0.00132494
AFF2	UP	Overlapped	1.62	0.001596947
LOC105377982	UP	No overlap	1.45	0.00281547
EPDR1	UP	Overlapped	0.82	0.002847942
NRCAM	UP	No overlap	1.95	0.002847942
LINC01852	UP	Overlapped	0.81	0.003231738
VLDLR-AS1	UP	Overlapped	2.07	0.00327304
C4orf19	UP	No overlap	0.62	0.003554836
TNFRSF10D	UP	No overlap	0.95	0.003606475
FHL1	UP	Overlapped	0.88	0.003747747
SAA1	UP	Overlapped	1.60	0.003747747
BMP2	UP	No overlap	0.87	0.004121004
MMP19	UP	Overlapped	0.88	0.004121004
MYMX	UP	Overlapped	1.21	0.004481529
LEP	UP	Overlapped	0.98	0.004609544
VLDLR	UP	Overlapped	1.03	0.004609544
HSPB8	UP	Overlapped	0.92	0.00495356
SYNC	UP	Overlapped	0.97	0.006621705
ARHGDIG	UP	Overlapped	1.83	0.006846737
SRPX2	UP	Overlapped	0.87	0.006846737
DZIP1L	UP	No overlap	0.61	0.007102318
MYZAP	UP	Overlapped	0.84	0.007470579
LYZ	UP	No overlap	1.44	0.008020398
LOC105376486	UP	No overlap	0.77	0.008411787
NQO1	UP	Overlapped	1.10	0.00877626
RHOXF1	UP	Overlapped	1.15	0.00877626
HSD11B1	UP	Overlapped	1.04	0.00907245
HOXC8	UP	No overlap	0.74	0.009694005
GFRAL	UP	No overlap	1.06	0.009995821
TRDN	UP	Overlapped	1.77	0.009995821
UPK1A	UP	No overlap	1.81	0.009995821
LOC107985524	UP	No overlap	0.80	0.010203662
MSR1	UP	Overlapped	0.85	0.0106403

LOC105377918	UP	Overlapped	1.28	0.010912143
ANKRD33B	UP	No overlap	0.97	0.011552178
DCUN1D3	UP	No overlap	0.62	0.011552178
PIR	UP	Overlapped	0.72	0.011701
BAG3	UP	Overlapped	0.81	0.011995574
TREM1	UP	Overlapped	1.23	0.011995574
NLRP3	UP	Overlapped	0.96	0.012694626
CSF3	UP	Overlapped	2.63	0.01359005
SELE	UP	Overlapped	2.20	0.01359005
LOC107987423	UP	Overlapped	1.26	0.013602757
SLC25A25	UP	No overlap	0.86	0.014003154
TNMD	UP	Overlapped	0.92	0.015506415
HS3ST2	UP	Overlapped	1.34	0.015543886
LOC105378685	UP	No overlap	0.95	0.016873594
STX11	UP	No overlap	0.64	0.017372013
LINC01402	UP	Overlapped	0.95	0.017686376
LOC105378915	UP	No overlap	1.06	0.019179403
PCLO	UP	No overlap	1.02	0.019179403
BHMT2	UP	Overlapped	0.79	0.019429999
LOC105373202	UP	No overlap	1.43	0.019436683
VENTX	UP	Overlapped	0.81	0.020262138
LOC100506178	UP	No overlap	0.98	0.020598872
LOC105372270	UP	Overlapped	1.15	0.020598872
SAA2	UP	Overlapped	1.54	0.020598872
TLCD2	UP	Overlapped	0.68	0.020598872
SLC2A3	UP	Overlapped	0.99	0.021041132
LOC105374166	UP	No overlap	1.40	0.021632068
DOK2	UP	Overlapped	0.89	0.022439888
GZMK	UP	Overlapped	1.17	0.022439888
RGS20	UP	No overlap	1.06	0.022439888
LOC105374127	UP	No overlap	1.06	0.022687485
PROK1	UP	Overlapped	1.72	0.022989648
ITSN1	UP	No overlap	0.61	0.023771542
CRYAB	UP	Overlapped	1.10	0.024551553
GRB14	UP	Overlapped	0.89	0.024620897
HSPA12A	UP	No overlap	0.85	0.025683373
LINC00857	UP	Overlapped	0.64	0.025683373
METRNL	UP	Overlapped	0.68	0.025683373
COL8A1	UP	No overlap	1.14	0.025789931
SH2D4A	UP	Overlapped	0.60	0.025841092
LOC101927243	UP	Overlapped	1.29	0.026055084
TNFSF14	UP	Overlapped	0.76	0.026055084
DPT	UP	Overlapped	1.01	0.026410262
WDPCP	UP	No overlap	0.69	0.02683696
NECAB1	UP	No overlap	0.93	0.027173205
CCL3	UP	Overlapped	1.33	0.031057866
MIR22HG	UP	Overlapped	0.67	0.031790483

CHST3	UP	No overlap	0.65	0.031830723
NRP2	UP	No overlap	0.70	0.032458335
PMEPA1	UP	Overlapped	0.78	0.03485418
EGFL6	UP	No overlap	2.54	0.036363136
HAS1	UP	Overlapped	1.86	0.036363136
NPR3	UP	Overlapped	1.17	0.036764165
PTPRQ	UP	No overlap	0.95	0.036764165
RNF122	UP	Overlapped	0.98	0.036764165
C19orf38	UP	Overlapped	0.97	0.037751557
PYGL	UP	No overlap	0.63	0.037751557
LINC00891	UP	No overlap	0.72	0.040569439
LOC100132741	UP	No overlap	0.72	0.040569439
ARHGEF39	UP	No overlap	0.60	0.0409044
ID4	UP	No overlap	0.70	0.041142481
LOC107984952	UP	No overlap	0.83	0.04420987
FGF18	UP	Overlapped	1.09	0.044338161
URAD	UP	Overlapped	2.60	0.044338161
ANKDD1A	UP	Overlapped	0.90	0.04489705
FERMT3	UP	Overlapped	0.63	0.046002361
GLYAT	UP	No overlap	0.75	0.046002361
LOC102724850	UP	Overlapped	1.00	0.046002361
LOC105374657	UP	No overlap	1.74	0.046002361
HMOX1	UP	Overlapped	0.80	0.046420871
MYL9	UP	Overlapped	0.76	0.04652077
CD209	UP	No overlap	0.76	0.046951076
FCGR2A	UP	Overlapped	0.64	0.048017337
C5orf64	UP	Overlapped	0.91	0.048407326
IL6	UP	Overlapped	1.75	0.049169961
RAB26	DN	Overlapped	-1.98	0.000601018
DEGS2	DN	No overlap	-1.88	0.00132494
TMEM25	DN	No overlap	-0.74	0.001596947
GRIK3	DN	No overlap	-1.82	0.001777693
TOX3	DN	Overlapped	-1.75	0.001777693
SALL2	DN	No overlap	-0.73	0.002164853
TOP2A	DN	No overlap	-1.57	0.002847942
TMEM132A	DN	No overlap	-1.55	0.004121004
WNK4	DN	No overlap	-1.80	0.004852878
VWA2	DN	No overlap	-1.18	0.005346739
BRIP1	DN	Overlapped	-0.86	0.005546229
CRB3	DN	No overlap	-0.98	0.005546229
GP2	DN	No overlap	-2.70	0.005546229
PCSK4	DN	No overlap	-1.10	0.006846737
PLEKHN1	DN	No overlap	-1.80	0.006846737
SLC26A3	DN	No overlap	-2.62	0.006846737
SOWAHB	DN	No overlap	-1.53	0.007470579
GALNT6	DN	No overlap	-1.75	0.00877626

WNT7B	DN	No overlap	-1.84	0.00907245
TMEM63C	DN	No overlap	-2.41	0.009370351
C6	DN	Overlapped	-1.55	0.009857074
NUSAP1	DN	No overlap	-0.88	0.009922929
PROM2	DN	No overlap	-1.63	0.009922929
ALOX15B	DN	No overlap	-2.64	0.009995821
CHIA	DN	Overlapped	-3.14	0.009995821
ESPN	DN	No overlap	-1.83	0.009995821
KIF20A	DN	Overlapped	-1.40	0.009995821
MEF2B	DN	Overlapped	-1.68	0.009995821
SEZ6L2	DN	No overlap	-1.27	0.009995821
FAM83E	DN	No overlap	-1.65	0.010235587
TMEM184A	DN	No overlap	-1.37	0.010235587
ANLN	DN	No overlap	-1.50	0.0106403
BUB1B	DN	No overlap	-1.62	0.0106403
LYPD6	DN	No overlap	-1.78	0.010912143
OPRPN	DN	No overlap	-3.28	0.010912143
C4B	DN	No overlap	-2.37	0.010972408
INPP5J	DN	No overlap	-1.64	0.011452538
KIFC1	DN	No overlap	-0.94	0.011788214
ALB	DN	No overlap	-1.75	0.011995574
LOC110091777	DN	No overlap	-1.24	0.011995574
PKIB	DN	No overlap	-1.57	0.013312259
TFAP2B	DN	No overlap	-2.08	0.014003154
ANKRD24	DN	Overlapped	-0.76	0.015883115
CELSR1	DN	No overlap	-1.43	0.015883115
RET	DN	No overlap	-1.24	0.015883115
SYT12	DN	No overlap	-1.36	0.015883115
EPPK1	DN	No overlap	-1.82	0.016568581
LMX1B	DN	No overlap	-1.52	0.016592444
CBLC	DN	No overlap	-1.86	0.017619312
ERVMER34-1	DN	No overlap	-1.38	0.017619312
ERBB2	DN	No overlap	-1.16	0.017686376
AGRN	DN	No overlap	-1.21	0.018307441
CLDN7	DN	No overlap	-1.26	0.018517761
RASSF7	DN	No overlap	-1.43	0.018910076
TFAP2A	DN	No overlap	-1.83	0.01900991
CHST8	DN	No overlap	-2.31	0.019179403
LOC285097	DN	No overlap	-1.60	0.019179403
RASEF	DN	No overlap	-1.97	0.019179403
TC2N	DN	No overlap	-1.12	0.019179403
SORD	DN	No overlap	-0.93	0.019429999
LINC01087	DN	No overlap	-2.38	0.020262138
SLC44A4	DN	No overlap	-1.80	0.020300591
ABAT	DN	No overlap	-0.65	0.020598872
TMEM26	DN	No overlap	-1.79	0.020598872
GXYLT1P3	DN	No overlap	-2.00	0.020952196

FOXA1	DN	No overlap	-2.31	0.021135679
NBPF6	DN	No overlap	-1.85	0.021135679
TSPY26P	DN	Overlapped	-0.91	0.021994315
EHD3	DN	No overlap	-0.73	0.022984467
WIPF3	DN	No overlap	-0.69	0.023225823
MAMDC4	DN	Overlapped	-1.39	0.02329485
SBK1	DN	No overlap	-1.33	0.02329485
BIRC5	DN	No overlap	-1.22	0.023494809
SSPOP	DN	Overlapped	-0.92	0.023494809
LYPD3	DN	No overlap	-1.70	0.023771542
MUC5B	DN	Overlapped	-2.02	0.023771542
TROAP	DN	Overlapped	-1.21	0.023771542
CAPN13	DN	No overlap	-1.96	0.023825093
PLA2G4F	DN	No overlap	-1.33	0.024548531
ZNF552	DN	No overlap	-0.93	0.024548531
CDSN	DN	No overlap	-1.39	0.024551553
CELSR2	DN	No overlap	-1.06	0.024620897
TTC36	DN	Overlapped	-1.23	0.024620897
CRACR2B	DN	No overlap	-1.10	0.025187412
CARD14	DN	No overlap	-1.10	0.025361594
SEC14L2	DN	No overlap	-1.48	0.025683373
ALDH3B2	DN	No overlap	-2.54	0.025841092
LOC112268037	DN	No overlap	-1.64	0.025841092
MACC1	DN	No overlap	-1.03	0.026055084
TMEM238	DN	No overlap	-1.57	0.026567758
GCK	DN	Overlapped	-1.20	0.026614808
S100P	DN	No overlap	-1.85	0.027116078
CATSPERB	DN	No overlap	-1.30	0.027173205
OBSCN	DN	Overlapped	-0.88	0.027203402
MAP3K9	DN	No overlap	-1.25	0.027366939
PSD3	DN	No overlap	-0.81	0.027366939
PLEKHS1	DN	Overlapped	-1.89	0.028990673
TGFB2	DN	No overlap	-1.03	0.02907288
INO80B	DN	No overlap	-0.67	0.029993641
CYP4F8	DN	No overlap	-2.15	0.030258612
LINC02076	DN	Overlapped	-0.88	0.030426568
ALCAM	DN	No overlap	-1.32	0.030498995
PPP1R14C	DN	Overlapped	-1.56	0.031539567
ELF3	DN	No overlap	-1.76	0.031830723
LLGL2	DN	No overlap	-0.99	0.031858175
HJURP	DN	No overlap	-0.95	0.03209884
KRT80	DN	Overlapped	-1.87	0.032540003
NECTIN1	DN	No overlap	-0.84	0.032915542
GGT1	DN	No overlap	-1.10	0.033741031
MYB	DN	No overlap	-1.62	0.033849256
IRS1	DN	No overlap	-0.63	0.034299023
AZGP1	DN	No overlap	-0.67	0.03467232

KRT8	DN	No overlap	-1.40	0.03467232
CDT1	DN	Overlapped	-1.23	0.034830469
CASZ1	DN	No overlap	-1.02	0.03520249
MGAM2	DN	Overlapped	-1.93	0.035220428
PI3	DN	Overlapped	-1.94	0.035220428
SPTBN2	DN	No overlap	-1.26	0.035220428
MYBL2	DN	Overlapped	-0.88	0.035429927
PLCH2	DN	No overlap	-1.53	0.036363136
NR6A1	DN	No overlap	-0.80	0.036463022
SLC27A6	DN	No overlap	-1.31	0.036463022
SYT7	DN	No overlap	-0.83	0.036463022
MCM2	DN	No overlap	-0.75	0.036795424
CYP2G1P	DN	No overlap	-1.70	0.037380271
PLS1	DN	No overlap	-1.34	0.037751557
PKP3	DN	No overlap	-1.52	0.038513017
PARD6B	DN	No overlap	-0.76	0.038762459
CHAD	DN	No overlap	-1.49	0.038860598
KCNC3	DN	No overlap	-1.08	0.039003705
EDN1	DN	No overlap	-0.73	0.039520174
IQCH	DN	No overlap	-0.83	0.03964113
EVPL	DN	No overlap	-1.33	0.040279869
IGFBP2	DN	No overlap	-1.00	0.040569439
SMIM22	DN	No overlap	-1.67	0.040569439
DTL	DN	No overlap	-1.14	0.041142481
LMTK3	DN	No overlap	-1.44	0.041142481
LOC105376436	DN	No overlap	-2.69	0.041142481
PTPRT	DN	No overlap	-1.59	0.041142481
SFN	DN	No overlap	-1.29	0.041142481
ST8SIA6	DN	No overlap	-1.21	0.041142481
SYNE4	DN	No overlap	-1.34	0.041142481
ZNF681	DN	No overlap	-0.60	0.041142481
KIF5C	DN	No overlap	-1.13	0.041214607
PNMT	DN	No overlap	-1.32	0.041214607
FAM83H	DN	No overlap	-1.06	0.04198391
RAB27B	DN	No overlap	-1.31	0.042508503
UHRF1	DN	Overlapped	-1.00	0.042508503
HEPACAM2	DN	No overlap	-2.38	0.043185538
TACSTD2	DN	No overlap	-1.43	0.043185538
CLDN3	DN	No overlap	-1.85	0.043359393
VTCN1	DN	No overlap	-2.04	0.044237625
PIANP	DN	No overlap	-0.77	0.044338161
PRR36	DN	No overlap	-1.60	0.044776449
S100A14	DN	No overlap	-1.69	0.044776449
ABCB9	DN	No overlap	-0.62	0.04487779
LRIG1	DN	Overlapped	-0.73	0.04487779
NCCRP1	DN	No overlap	-1.08	0.04489705
GATA3	DN	No overlap	-1.03	0.045439803

MYO5B	DN	No overlap	-1.25	0.045654073
CDC45	DN	No overlap	-1.13	0.046002361
MELTF	DN	No overlap	-0.97	0.046002361
RAD54L	DN	No overlap	-0.95	0.046002361
NEK10	DN	No overlap	-1.48	0.046197395
AZGP1P1	DN	Overlapped	-1.22	0.04652077
KRT6B	DN	No overlap	-1.70	0.046652481
RORC	DN	No overlap	-1.14	0.046951076
LZTS1	DN	No overlap	-0.83	0.046983949
CENPF	DN	No overlap	-0.87	0.047792522
ST14	DN	No overlap	-1.31	0.048017337
CACNG4	DN	No overlap	-1.81	0.048030104
LOC105377211	DN	No overlap	-0.72	0.048242403
USP43	DN	No overlap	-1.69	0.048242403
DNAJC12	DN	No overlap	-1.44	0.048846258
SLC7A3	DN	No overlap	-1.52	0.049494752
MKI67	DN	No overlap	-1.12	0.049937226
